# Assessment of Knowledge, Attitudes, and Practices Related to the Hall Technique Among Pediatric Dentists in Jordan

**DOI:** 10.7759/cureus.113679

**Published:** 2026-07-30

**Authors:** Alaa O Almaaita, Areej Alshdefat, Hind Almaaitah, Heba Altarawneh, Saeda Aloran, Oraib Al-Ababneh, Eman Hammouri

**Affiliations:** 1 Dentistry, Jordanian Royal Medical Services, Amman, JOR; 2 Pediatric Dentistry, Jordanian Royal Medical Services, Amman, JOR

**Keywords:** attitudes, hall technique, jordan, knowledge, pediatric dentistry, practice

## Abstract

Objectives: The Hall technique (HT) manages dental caries in primary molars by sealing the lesion with a preformed metal crown, a minimally invasive strategy aimed at arresting the disease process. Although convincing evidence of efficacy is available, global acceptance varies. This study aimed to evaluate the knowledge, attitudes, and practices (KAP) of Jordanian pediatric dentists regarding the HT and its main features.

Methods: A cross-sectional study was carried out using an online questionnaire distributed to Jordanian pediatric dentists from November 1 to 30, 2025. The questionnaire surveyed demographics, knowledge (rationale, indications), attitudes (efficiency, evidence-based), and practices (current use, challenges, concerns). Descriptive statistics, chi-square tests, and binomial logistic regression were used to identify predictors of HT implementation among 120 participants.

Results: Most respondents were female (103/120, 85.8%) and board-certified (91/120, 75.8%). The rationale for caries arrest by sealing with the HT was understood by 118/120 (98.3%) of the respondents. The frequency of HT use was 104/120 (86.7%). Years of clinical practice were significantly associated with the perception that HT is best for uncooperative patients (p = 0.031), and belief in medical contraindications was associated with reduced HT use (p = 0.024). The main concerns were occlusion (65/120, 54.2%), sealing over caries (54/120, 45.0%), and parental acceptance (54/120, 45.0%). The majority perceived HT as a time-saving method (111/120, 92.5%), and the attitudes were generally positive. Logistic regression analysis revealed that the belief in time-saving (OR = 8.58, 95% CI: 2.09-35.21, p = 0.003) and evidence base (OR = 5.21, 95% CI: 1.37-19.81, p = 0.015) were significant positive predictors for HT use, whereas the concern of parental acceptance (OR = 0.24, 95% CI: 0.09-0.65, p = 0.005) and sealing over caries (OR = 0.34, 95% CI: 0.13-0.89, p = 0.027) were significant negative predictors. The model explained 28.4-45.2% of the variance in HT use and was a good fit (Hosmer-Lemeshow p = 0.342).

Conclusion: Pediatric dentists in Jordan had a high level of knowledge of and use of the HT, with adoption rates higher than in many other countries. A positive perception of time efficiency and evidence-based belief strongly favor clinical use, while concerns about parental acceptance and sealing over caries are the main barriers to consistent implementation.

## Introduction

Dental caries in primary teeth remains a major public health problem worldwide, affecting millions of children and their quality of life, nutritional status, and future oral health [1]. The management of carious primary molars has evolved from traditional methods based on complete caries removal and conventional restorations to minimally invasive, biologically driven strategies that aim to preserve tooth structure and arrest disease progression [2]. The Hall technique (HT) represents a significant, evidence-based advancement in the management of carious primary molars [3]. The technique involves cementing a preformed metal crown onto a carious tooth without local anesthesia administration, caries removal, or tooth preparation [4]. The fundamental biological principle underpinning HT is that complete and permanent sealing of carious lesions from the oral environment disrupts the biofilm ecosystem, depriving cariogenic bacteria of substrates and arresting the disease process through environmental modification rather than surgical excision [5].

Strong evidence from systematic reviews and randomized controlled trials demonstrates that HT achieves high clinical success rates (exceeding 90% at two to five years), superior longevity compared with traditional restorations, excellent cost-effectiveness, and high acceptability among both children and parents [6,7]. In spite of this strong evidence base, the uptake of HT in pediatric dentistry varies widely between countries and clinical settings [8]. This variation is attributed to multiple factors, including differences in postgraduate training curricula, entrenched clinical philosophies, practitioner beliefs, and perceptions of patient and parent acceptance [9,10].

Jordan possesses a well-developed dental care infrastructure with a strong emphasis on specialist training. However, the extent to which Jordanian pediatric dentists have adopted this evidence-based, minimally invasive approach has not been systematically evaluated. Understanding the current landscape of knowledge, attitudes, and practices (KAP) related to HT is essential for identifying gaps, guiding educational initiatives, and ultimately facilitating broader access to effective, patient-centered caries management for Jordanian children.

The primary objective of this study was to comprehensively assess the KAP of pediatric dentists in Jordan regarding the HT. The secondary objective was to identify independent predictors of HT adoption through multivariable regression modeling, thereby informing targeted educational interventions and curriculum development in pediatric dentistry training programs.

## Materials and methods

Ethics statement

This study received ethical approval from the Institutional Review Board (IRB) of the Jordanian Royal Medical Services on November 3, 2025 (registration number: 10_15/2025). Final authorization for dissemination was granted by the Educational and Training Directorate of the Jordanian Royal Medical Services on January 23, 2026. The study was conducted in accordance with the ethical principles outlined in the Declaration of Helsinki. Electronic informed consent was obtained from all participants prior to survey completion.

Study design and setting

A cross-sectional study was conducted over a one-month period from November 1 to 30, 2025. The study targeted pediatric dentists practicing in Jordan across multiple sectors, including hospital-based services, the Ministry of Health, university-affiliated dental centers, and private practices. The study is reported in accordance with the Strengthening the Reporting of Observational Studies in Epidemiology (STROBE) statement for cross-sectional studies [11].

Study population

The target population comprised all qualified pediatric dentists currently practicing in Jordan. Inclusion criteria were (1) holding a recognized postgraduate qualification in pediatric dentistry (board certified, PhD, or equivalent); (2) currently practicing in Jordan during the study period; and (3) willing to provide electronic informed consent. Exclusion criteria were (1) general dentists without specialist training and (2) incomplete responses (<80% of survey items completed).

Recruitment strategy

Participants were identified through the Jordanian Dental Association's specialist registry and institutional databases from the Royal Medical Services, Ministry of Health, and university-affiliated dental centers. Invitations were sent via email with a unique survey link and followed up with two reminder emails at two-week intervals. The survey was accessible for a four-week period from November 1 to 30, 2025. Of 180 eligible pediatric dentists identified through the sampling frame, 140 consented to participate, and 120 completed the survey in full, yielding a response rate of 66.7% (120/180) and a completion rate of 85.7% (120/140) among consenting participants. Non-participation was primarily due to lack of response to the invitation (n = 30) or incomplete responses (n = 10). The sampling frame included all registered pediatric dentistry specialists in Jordan, though the final sample predominantly comprised participants from the Royal Medical Services (68.3%), reflecting the concentration of specialist services within this tertiary referral system. A total of 120 eligible pediatric dentists completed the survey, representing the final analytical cohort.

Survey instrument

Data were collected using a structured, self-administered online questionnaire. The survey instrument was developed through literature review [9,10], expert validation by two pediatric dentistry specialists and one biostatistician, and pilot testing on a small sample of dentists (n = 5). The final questionnaire comprised four sections: (1) demographics and professional characteristics; (2) knowledge assessment (rationale, indications, and contraindications); (3) attitude assessment using a five-point Likert scale (1 = strongly disagree to 5 = strongly agree); and (4) practice assessment (current use, frequency, concerns, and interest in training).

Psychometric Properties of the Attitude Scale

To establish the internal consistency of the attitude scale (Section C, 10 Likert items), Cronbach's alpha was calculated. The scale demonstrated acceptable internal consistency (α = 0.81), indicating satisfactory reliability for the attitude construct. Content validity was assessed by the expert panel, with item-level content validity index (I-CVI) values ranging from 0.83 to 1.00 and a scale-level content validity index (S-CVI/Ave) of 0.92, indicating excellent content validity. Additionally, exploratory factor analysis using principal component analysis with varimax rotation was performed to confirm the underlying factor structure. The Kaiser-Meyer-Olkin (KMO) measure of sampling adequacy was 0.78, and Bartlett's test of sphericity was significant (χ² = 287.6, df = 45, p < 0.001), confirming the appropriateness of factor analysis. Two factors with eigenvalues >1.0 were extracted, explaining 58.7% of the total variance. These factors were interpreted as (1) "Evidence-Based Belief and Efficiency" and (2) "Clinical Utility and Training Interest". Detailed factor loadings are presented in the Results section.

Statistical analysis

All statistical analyses were performed using IBM SPSS Statistics for Windows, Version 25.0 (IBM Corp., Armonk, NY) and R software version 4.2.2 (R Foundation for Statistical Computing, Vienna, Austria) with the *logistf* package for penalized logistic regression. A two-tailed p-value <0.05 was considered statistically significant. Categorical variables are presented as frequencies and percentages (n, %). Associations between demographic variables and KAP outcomes were examined using chi-square (χ²) tests or Fisher's exact tests as appropriate.

Given the small number of non-users (n = 16) relative to the number of predictors, we employed Firth's penalized maximum likelihood logistic regression to reduce bias and address the rare events concern [12]. Variables with p < 0.10 in bivariate analysis or strong theoretical relevance were entered into the model using the enter method. Although occlusion was the most frequently cited, bivariate analysis found no significant association with HT use (p = 0.412), so it was excluded from the multivariable model to maintain parsimony and avoid overfitting, given the limited number of non-users (n = 16). Bootstrap-corrected confidence intervals (1000 resamples) were calculated for all predictors to provide more robust uncertainty estimates.

Model performance was evaluated using multiple metrics. The Hosmer-Lemeshow goodness-of-fit test was used to assess model calibration (p > 0.05 indicates adequate calibration). Pseudo-R² values (Cox & Snell: 28.4%; Nagelkerke: 45.2%) are reported to provide an indication of the proportion of variance explained by the model. The discrepancy between these values reflects the limitations of pseudo-R² statistics in logistic regression, where they are influenced by the baseline model and should not be interpreted as exact analogs to linear regression R². The Akaike information criterion (AIC) and Bayesian information criterion (BIC) were calculated to assess model parsimony (AIC = 112.4; BIC = 128.7). To evaluate model generalizability, 10-fold cross-validation was performed, with the mean cross-validated area under the curve (AUC) reported (AUC = 0.86, 95% CI: 0.81-0.91). Results are presented as adjusted odds ratios (OR) with 95% confidence intervals (CI). With 104 users and 16 non-users, and up to six predictors in the final model, the use of penalized regression and bootstrap validation mitigates the risk of overfitting and ensures the stability of the estimates.

## Results

Demographic and professional characteristics

Table 1 presents the demographic and professional profile of the 120 respondents. The sample was predominantly female (85.8%, n = 103). The median age category was 36-40 years (30.0%, n = 36). The majority held board certification (75.8%, n = 91). The Royal Medical Services was the primary training institution for most participants (68.3%, n = 82). Clinical experience was broadly distributed across experience categories.

**Table 1:** Demographic and professional characteristics of the participants (N = 120). This table presents the demographic and professional profile of the 120 Jordanian pediatric dentists who participated in the study. Data are presented as frequencies (n) and percentages (%). The sample was predominantly female (103/120, 85.8%), with the largest age group being 36-40 years (36/120, 30.0%). The majority held board certification (91/120, 75.8%) and trained at the Royal Medical Services (82/120, 68.3%). Years of clinical experience were broadly distributed across categories, with the largest proportion having 10-14 years of experience (35/120, 29.2%). Abbreviations: N, total sample size; n, frequency; %, percentage; MSc, Master of Science; PhD, Doctor of Philosophy.

Variable	Category	n	%
Gender	Female	103	85.8
	Male	17	14.2
Age group	24-30 years	31	25.8
	31-35 years	23	19.2
	36-40 years	36	30.0
	41-45 years	21	17.5
	46-50 years	6	5.0
	>50 years	3	2.5
Highest qualification	Board certificate	91	75.8
	MSc	24	20.0
	PhD	1	0.8
	Not specified	4	3.3
Primary training institution	Royal Medical Services	82	68.3
	University	16	13.3
	Ministry of Health	13	10.8
	Abroad	9	7.5
Years of clinical experience	<5 years	28	23.3
	5-9 years	31	25.8
	10-14 years	35	29.2
	≥15 years	26	21.7

Knowledge and practice

Table 2 summarizes knowledge and practice patterns. Knowledge levels were exceptionally high, with nearly all respondents (98.3%, n = 118) correctly identifying the core rationale of HT as "arresting caries by sealing." The current utilization rate of HT was 86.7% (n = 104). The main concerns identified were occlusal issues (54.2%, n = 65), sealing over caries (45.0%, n = 54), and parental acceptance (45.0%, n = 54).

**Table 2:** Knowledge and practices related to the Hall technique (N = 120). This table summarizes knowledge levels and clinical practices regarding the Hall technique (HT) among Jordanian pediatric dentists. Knowledge of the core rationale (arresting caries by sealing) was nearly universal (118/120, 98.3%). Current HT utilization was 86.7% (104/120). The primary indication identified was asymptomatic carious primary molars (115/120, 95.8%). A majority of respondents believed medical contraindications to HT exist (78/120, 65.0%). The main concerns identified were occlusion (65/120, 54.2%), sealing over caries (54/120, 45.0%), and parental acceptance (54/120, 45.0%). Percentages for indications and concerns exceed 100% as multiple responses were permitted. Abbreviations: N, total sample size; n, frequency; %, percentage; HT, Hall technique.

Variable	Category	n	%
Current HT user	Yes	104	86.7
	No	16	13.3
Main rationale (correct)	Arrest caries by sealing	118	98.3
Primary indication	Asymptomatic carious primary molar	115	95.8
	Conventional restoration not possible	38	31.7
Believes medical contraindications exist	Yes	78	65.0
	No	42	35.0
Main concerns	Occlusion	65	54.2
	Sealing over caries	54	45.0
	Parental acceptance	54	45.0

Attitudes toward the Hall technique

Table 3 presents attitude assessments. The majority perceived HT as time-saving (92.5%, n = 111), best for uncooperative patients (85.8%, n = 103), and evidence-based (88.3%, n = 106). A large majority (85.0%, n = 102) expressed interest in additional HT training.

**Table 3:** Attitudes toward the Hall technique (N = 120). This table presents the attitudes of Jordanian pediatric dentists toward the Hall technique (HT) using dichotomous (yes/no) and Likert scale responses. The majority perceived HT as time-saving (111/120, 92.5%), believed it is best for uncooperative patients (103/120, 85.8%), and considered it evidence-based (106/120, 88.3%). A large majority expressed interest in additional HT training (102/120, 85.0%). Abbreviations: N, total sample size; n, frequency; %, percentage; HT, Hall technique.

Variable	Response	n	%
Believes HT is time-saving	Yes	111	92.5
Believes HT is best for uncooperative patients	Yes	103	85.8
Believes HT is evidence-based	Agree/Strongly agree	106	88.3
Interested in further training	Yes	102	85.0

Reliability and Factor Analysis of the Attitude Scale

The attitude scale (Section C, 10 Likert items) demonstrated acceptable internal consistency, with a Cronbach's alpha of 0.81, indicating good reliability for the attitude construct. Exploratory factor analysis using principal component analysis with varimax rotation revealed a two-factor structure, explaining 58.7% of the total variance. The KMO measure of sampling adequacy was 0.78, and Bartlett's test of sphericity was significant (χ² = 287.6, df = 45, p < 0.001), confirming the appropriateness of factor analysis. Table 4 presents the factor loadings for each item.

**Table 4:** Factor loadings of attitude scale items (varimax rotation). This table presents the factor loadings of the 10 attitude scale items following exploratory factor analysis with varimax rotation. Two factors were extracted, explaining 58.7% of the total variance. Factor 1, "Evidence-Based Belief and Efficiency," comprises items related to the scientific foundation and time-saving nature of HT. Factor 2, "Clinical Utility and Training Interest," includes items related to clinical applicability and educational needs. The Kaiser-Meyer-Olkin (KMO) measure was 0.78, and Bartlett's test of sphericity was significant (χ² = 287.6, df = 45, p < 0.001). Cronbach's alpha for the scale was 0.81. Abbreviations: HT, Hall technique; KMO, Kaiser-Meyer-Olkin; χ², chi-square; df, degrees of freedom; p, p-value.

Item	Factor 1: Evidence-Based Belief and Efficiency	Factor 2: Clinical Utility and Training Interest
HT is evidence-based	0.82	0.18
HT is time-saving	0.78	0.22
HT is biologically sound	0.75	0.25
HT is cost-effective	0.71	0.29
HT should be taught in training	0.68	0.34
HT is best for uncooperative patients	0.22	0.79
HT is acceptable to parents	0.18	0.76
Interested in further training	0.15	0.72
Confident in performing HT	0.35	0.68
HT is a valid treatment option	0.42	0.61
Eigenvalue	3.42	2.45
% of variance explained	34.2%	24.5%
Cumulative %	34.2%	58.7%

Bivariate associations

Table 5 presents bivariate analyses. Years of experience was significantly associated with belief that HT is best for uncooperative patients (p = 0.031). Belief in medical contraindications was significantly associated with lower HT use (p = 0.024). Place of training and years of experience showed no significant association with current HT use.

**Table 5:** Inferential statistics: Associations between variables. This table presents the results of bivariate analyses examining associations between demographic/professional variables and key knowledge, attitudes, and practices (KAP) outcomes regarding the Hall technique (HT). Chi-square tests were used to assess associations. Significant associations were found between years of clinical experience and the belief that HT is best for uncooperative patients (p = 0.031), and between belief in medical contraindications and lower current HT use (p = 0.024). Place of training and years of experience showed no significant association with current HT use (p = 0.384 and p = 0.207, respectively). A p-value <0.05 was considered statistically significant. Abbreviations: HT, Hall technique; p, p-value.

Independent variable	Dependent variable	Statistical test	Test statistic	p-value
Years of experience	Believes HT is best for uncooperative patients	Chi-square (χ²)	8.94	0.031
Belief in medical contraindications	Current use of HT	Chi-square (χ²)	5.12	0.024
Place of training	Current use of HT	Chi-square (χ²)	3.04	0.384
Years of experience	Current use of HT	Chi-square (χ²)	4.63	0.207

Multivariable logistic regression

Table 6 presents the results of binomial logistic regression predicting current HT use. The overall model was statistically significant (χ² = 35.12, df = 6, p < 0.001), explaining 28.4% (Cox & Snell R²) to 45.2% (Nagelkerke R²) of the variance in HT use status. The Hosmer-Lemeshow goodness-of-fit test indicated good model calibration (χ² = 8.92, df = 8, p = 0.342). Model parsimony was supported by an AIC of 112.4 and a BIC of 128.7. Ten-fold cross-validation yielded a mean cross-validated AUC of 0.86 (95% CI: 0.81-0.91), indicating good generalizability of the model.

**Table 6:** Logistic regression predicting current Hall technique use. This table presents the results of binomial logistic regression analysis identifying independent predictors of current Hall technique (HT) use among Jordanian pediatric dentists (N = 120). The overall model was statistically significant (χ² = 35.12, df = 6, p < 0.001), explaining 28.4% (Cox & Snell R²) to 45.2% (Nagelkerke R²) of the variance in HT use status. The Hosmer-Lemeshow goodness-of-fit test indicated good model calibration (χ² = 8.92, df = 8, p = 0.342). Model parsimony was supported by an AIC of 112.4 and a BIC of 128.7. Ten-fold cross-validation yielded a mean cross-validated AUC of 0.86 (95% CI: 0.81-0.91), indicating good generalizability. Firth's penalized logistic regression was employed to address rare events bias. Bootstrap-corrected confidence intervals (1000 resamples) are reported for the original model estimates. Abbreviations: N, total sample size; HT, Hall technique; OR, odds ratio; CI, confidence interval; AIC, Akaike information criterion; BIC, Bayesian information criterion; AUC, area under the curve; χ², chi-square; df, degrees of freedom; R², coefficient of determination; p, p-value.

Predictor variable	OR	95% CI (Bootstrap-corrected)	Penalized OR	95% CI (Penalized)	Statistical test	Test statistic (Wald χ²)	p-value
Attitude: Time-saving	8.58	2.09-35.21	7.92	1.98-31.67	Wald χ²	8.74	0.003
Belief: HT is evidence-based	5.21	1.37-19.81	4.87	1.28-18.54	Wald χ²	5.92	0.015
Concern: Parental acceptance	0.24	0.09-0.65	0.27	0.10-0.72	Wald χ²	7.85	0.005
Concern: Sealing over caries	0.34	0.13-0.89	0.37	0.14-0.95	Wald χ²	4.89	0.027
Years of experience (≥10 years)	2.25	0.72-7.01	2.12	0.68-6.58	Wald χ²	1.95	0.163
Belief in medical contraindications	0.49	0.17-1.42	0.52	0.18-1.48	Wald χ²	1.71	0.191

Positive predictors (factors associated with higher odds of HT use) were time-saving perception (OR = 8.58, 95% CI: 2.09-35.21, Wald χ² = 8.74, p = 0.003) and evidence-based belief (OR = 5.21, 95% CI: 1.37-19.81, Wald χ² = 5.92, p = 0.015).

Negative predictors (factors associated with lower odds of HT use) were concern about parental acceptance (OR = 0.24, 95% CI: 0.09-0.65, Wald χ² = 7.85, p = 0.005) and concern about sealing over caries (OR = 0.34, 95% CI: 0.13-0.89, Wald χ² = 4.89, p = 0.027).

Non-significant predictors were years of experience ≥10 years (OR = 2.25, 95% CI: 0.72-7.01, Wald χ² = 1.95, p = 0.163) and belief in medical contraindications (OR = 0.49, 95% CI: 0.17-1.42, Wald χ² = 1.71, p = 0.191).

Positive predictors (promoters) were time-saving perception (OR = 8.58, p = 0.003) and evidence-based belief (OR = 5.21, p = 0.015).

**Figure 1:**
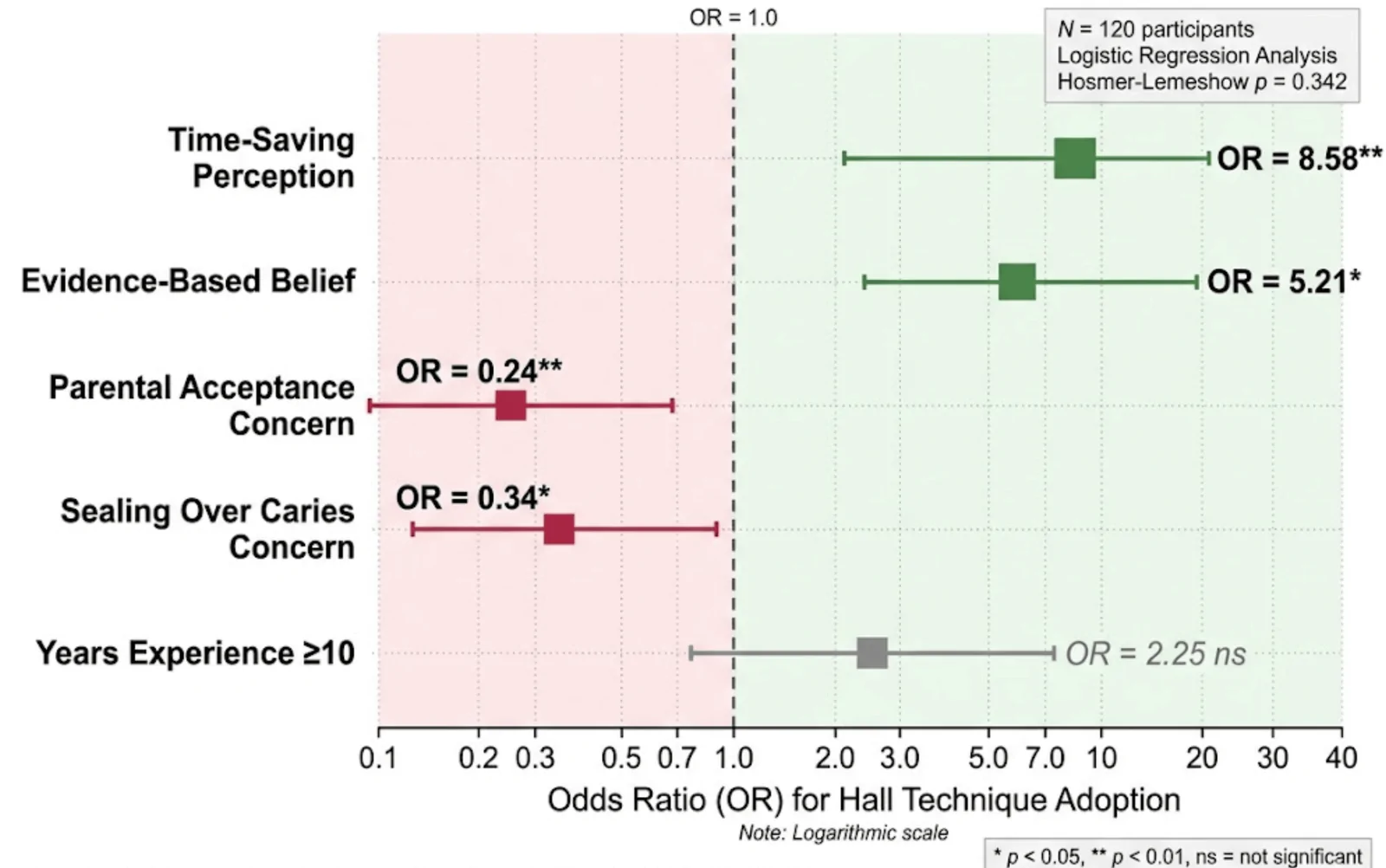
Forest plot of predictors for the adoption of Hall technique. Odds ratios (OR) and 95% confidence intervals (CI) from multivariable logistic regression (N = 120). The vertical dashed line at OR = 1.0 represents no effect. Points to the right indicate factors that increase the odds of HT use (promoters); points to the left indicate factors that decrease the odds of HT use (barriers). Horizontal lines represent 95% CIs; crossing the vertical line indicates non-significance. Green squares represent significant positive predictors (time-saving perception, evidence-based belief). Red squares represent significant negative predictors (parental acceptance concern, sealing over caries concern). Gray squares represent non-significant predictors (years of experience ≥10, belief in medical contraindications). Statistical tests used: Wald χ² test for individual predictors and Hosmer-Lemeshow χ² test for model fit. * p < 0.05, ** p < 0.01. Abbreviations: N, total sample size; OR, odds ratio; CI, confidence interval; HT, Hall technique; χ², chi-square; p, p-value; ns, not significant.

**Figure 2:**
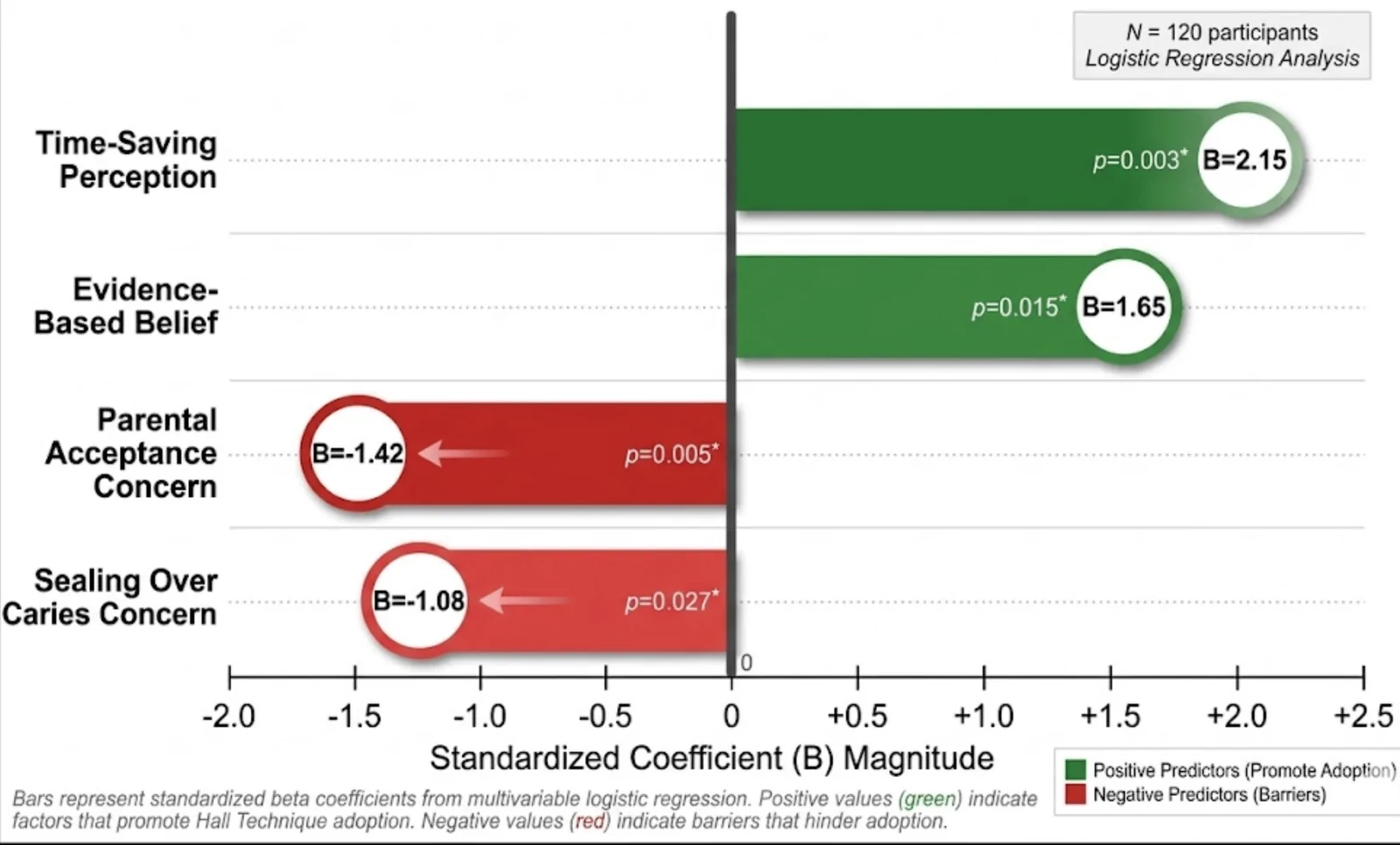
Standardized coefficient magnitude and direction of predictors. Standardized coefficient magnitude and direction of predictors for Hall technique adoption. Bar length corresponds to the absolute value of the standardized coefficient (B), indicating effect magnitude. Bars extending to the right (positive B values) represent factors that promote HT adoption (time-saving perception, evidence-based belief). Bars extending to the left (negative B values) represent factors that hinder HT adoption (parental acceptance concern, sealing over caries concern). Statistical test used: Wald χ² test from multivariable logistic regression. * p < 0.05, ** p < 0.01. Abbreviations: HT, Hall technique; B, standardized coefficient; χ², chi-square; p, p-value.

Negative predictors (barriers) were concern about parental acceptance (OR = 0.24, p = 0.005) and concern about sealing over caries (OR = 0.34, p = 0.027) (Figures 1, 2).

## Discussion

This study presents the first comprehensive evaluation of KAP regarding the HT among pediatric dentists in Jordan. Results show exceptionally high awareness, positive attitudes, and clinical application, with an overall utilization rate of 86.7% (104/120), which is comparable to or exceeds rates reported in several other countries [9,13].

Adoption rates and international comparison

The HT utilization rate of 86.7% among Jordanian pediatric dentists is notably higher than the rates reported in similar studies. In a survey of Saudi pediatric dentists, the uptake rates were lower (approximately 60-70%), primarily due to insufficient training exposure and concerns related to caries sealing [9]. Studies from the United Kingdom have reported adoption rates ranging from 45% to 65% among general dental practitioners, though these studies were conducted over seven years ago and may not reflect current practice patterns [14]. More recent surveys from North America and Europe suggest adoption rates between 50% and 75%, with significant variability based on training background and access to continuing education [10,13]. The higher adoption rate observed in our Jordanian cohort likely reflects the successful dissemination of this minimally invasive technique through structured postgraduate training programs, particularly within the Royal Medical Services system, as well as a strong emphasis on evidence-based practice in the local pediatric dentistry community.

However, direct cross-country comparisons should be interpreted with caution, as differences in study populations, healthcare systems, training curricula, and survey methodologies may contribute to observed variations. Standardized, contemporaneous multinational surveys are needed to more accurately benchmark adoption rates across different healthcare contexts.

Factors associated with HT adoption

Time-saving perception emerged as the strongest factor associated with adoption (OR = 8.58). This finding suggests that practical efficiency considerations in busy clinical environments serve as primary motivators for technique adoption. Jordanian pediatric dentists practice in settings characterized by high patient volumes and pressure to optimize clinical throughput. HT offers substantial time savings by eliminating local anesthesia administration, caries removal, and tooth preparation. This aligns with broader literature on technology adoption in healthcare, where workflow efficiency consistently emerges as a strong factor associated with adoption [15].

Belief that HT is evidence-based was the second strongest factor associated with adoption (emphasizing the value/importance of scientific rationale in clinical decision-making among Jordanian practitioners). This reflects a strong commitment to evidence-based practice principles and suggests that educational efforts highlighting the robust evidence base for HT may be effective in promoting adoption.

Barriers associated with lower HT adoption

Concern about parental acceptance was the strongest factor associated with lower odds of HT use (OR = 0.24), reducing the odds by 76%. This finding indicates that anticipated parental resistance exerts a powerful influence on clinical decision-making, potentially outweighing clinical considerations. This barrier has been consistently identified in international literature [10,15]. Research on actual parental acceptance suggests that concerns may be overestimated; when the rationale is clearly explained, most parents accept the approach [7].

Concern about sealing over active caries was associated with a 66% reduction in odds of use (OR = 0.34), reflecting the enduring psychological challenge of transitioning from a surgical to a biological paradigm. This concern persists despite robust evidence demonstrating that complete caries removal is unnecessary for disease arrest when the lesion is hermetically sealed [5].

Training interest

The substantial majority (85.0%) expressing interest in additional HT training represents an important opportunity for targeted educational interventions addressing identified barriers. This high level of training interest was observed despite a high current utilization rate (86.7%). At face value, the result appears contradictory; however, it is likely that the training interest among current users reflects a desire for advanced clinical skills rather than basic technique training. Specifically, these practitioners may be seeking education on handling complex or difficult cases; managing complications (e.g., crown loss, occlusal issues, and caries progression); optimizing crown selection and fitting; improving parent communication to address concerns about parental acceptance; and refining patient selection criteria. The survey item was deliberately broad - "interested in further HT training" - and did not differentiate between basic and advanced training needs. This limitation highlights the need for future surveys to distinguish between introductory and advanced educational priorities, in order to better tailor continuing professional development programs for pediatric dentists at different stages of clinical experience.

Knowledge assessment and ceiling effect

Knowledge levels regarding the core rationale of the HT were exceptionally high, with 98.3% of respondents correctly identifying the biological principle as "arresting caries by sealing." This finding reflects a strong foundational awareness of the technique's mechanism among Jordanian pediatric dentists. However, this high correct response rate suggests a significant ceiling effect in our knowledge assessment, raising questions about whether the item measured depth of clinical understanding or basic recognition. It is possible that the knowledge question was insufficiently discriminating, particularly given that the HT rationale is widely disseminated in pediatric dentistry literature and postgraduate training curricula. The current utilization rate of 86.7% suggests that knowledge is not the primary barrier to adoption, which is consistent with our finding that knowledge was not an independent predictor of HT use in the multivariable model. Nonetheless, the ceiling effect limits our ability to fully explore the relationship between deeper knowledge dimensions and clinical practice. Future studies should employ more granular knowledge assessments, including case-based scenarios, clinical vignettes, and questions addressing clinical reasoning, contraindications, patient selection, and complication management, to better capture the full spectrum of knowledge and its relationship to practice.

Limitations

This study has several limitations that readers should consider when interpreting the findings. First, the use of convenience sampling may limit the generalizability of the results to the entire population of pediatric dentists in Jordan, although the high response rate and representation from multiple practice sectors partially mitigate this concern. Notably, a significant proportion of participants (68.3%) were affiliated with the Royal Medical Services, reflecting the concentration of specialist pediatric dentistry services within this tertiary referral system. This sectoral predominance may limit the representativeness of findings for pediatric dentists in private practice, university-affiliated centers, or other public health sectors. Second, the cross-sectional design captures associations at a single time point but cannot establish causality or temporal relationships between the identified predictors and HT adoption. Therefore, all identified predictors should be interpreted as associations rather than determinants of HT adoption. Third, reliance on self-reported data may introduce social desirability bias, where respondents may over-report their knowledge and utilization of HT to align with perceived professional expectations. Fourth, the relatively small number of non-users (n = 16) limits the statistical precision of some estimates and may reduce the power to detect additional predictors of non-adoption. To address this, we employed Firth's penalized logistic regression and bootstrap-corrected confidence intervals (1000 resamples) to ensure the stability and reliability of our odds ratio estimates. Fifth, the study was conducted within a single country, which may limit the applicability of findings to other cultural and healthcare contexts where training, patient expectations, and practice environments differ. Sixth, the survey instrument, while validated by experts and pilot-tested, was not formally psychometrically validated in independent populations prior to this study, which may affect the comparability of findings across studies. Seventh, the knowledge assessment demonstrated a significant ceiling effect (98.3% correct for the core rationale), suggesting that the item may have measured basic recognition rather than depth of clinical understanding. Eighth, the survey did not differentiate between basic and advanced training needs, which may have limited the specificity of our assessment of training interest. Ninth, the online survey format may have excluded dentists with limited internet access or those less engaged with digital platforms, potentially introducing selection bias. Tenth, the study did not assess long-term clinical outcomes or patient-level factors, which could provide additional insights into barriers and facilitators to HT adoption from the patient and parent perspective. Future multicenter studies across different regions and healthcare systems, employing standardized and psychometrically validated instruments and incorporating objective measures of clinical practice, are warranted to further explore the factors influencing HT adoption and to develop targeted interventions addressing the identified barriers.

## Conclusions

Jordanian pediatric dentists demonstrate high knowledge and utilization of the HT, with adoption rates comparable to or exceeding those reported in many other countries. Positive perceptions regarding time efficiency and evidence base are strongly associated with clinical use, while concerns about parental acceptance and sealing over caries represent the main barriers associated with lower adoption. Targeted educational interventions addressing these specific barriers could further optimize HT adoption.

The high level of training interest (85.0%) underscores a clear demand for continuing professional development in HT. Educational programs should consider offering both basic and advanced modules to accommodate practitioners at different stages of clinical experience, with advanced modules focusing on complex case management, complication prevention, and patient communication strategies. Given the cross-sectional design of this study, the identified predictors should be interpreted as associations rather than determinants of HT adoption. Future longitudinal and multicenter studies are warranted to establish causal relationships and further explore the factors influencing HT adoption across different healthcare contexts.

## Appendices

Survey questionnaire

The following table presents the survey questionnaire used in this study. The instrument was developed by the authors through a literature review, expert validation by two pediatric dentistry specialists and one biostatistician, and pilot testing on a small sample of dentists (n = 5).

Survey Questionnaire: Assessment of Knowledge, Attitude, and Practice of the Hall Technique Among Pediatric Dentists in Jordan

**Table 7:** Section A: Demographic and professional characteristics.

Item	Question	Response options
A1	Gender	☐ Male ☐ Female
A2	Age group	☐ 24–30 years ☐ 31–35 years ☐ 36–40 years ☐ 41–45 years ☐ 46–50 years ☐ >50 years
A3	Highest qualification	☐ Board Certificate ☐ MSc ☐ PhD ☐ Other: ________
A4	Primary training institution	☐ Royal Medical Services ☐ University ☐ Ministry of Health ☐ Abroad ☐ Other: ________
A5	Years of clinical experience	☐ <5 years ☐ 5–9 years ☐ 10–14 years ☐ ≥15 years
A6	Current primary practice setting	☐ Royal Medical Services ☐ Ministry of Health ☐ University-affiliated Center ☐ Private Practice ☐ Other: ________

**Table 8:** Section B: Knowledge of the Hall technique.

Item	Question	Response options
B1	What is the Hall technique?	☐ Technique for placing stainless steel crowns after complete caries removal ☐ Technique involving cementing a preformed metal crown onto a carious primary molar without local anesthesia, caries removal, or tooth preparation ☐ Technique for restoring primary molars with composite resin ☐ Technique for extracting carious primary molars ☐ I am not familiar with this technique
B2	What is the primary biological rationale for the Hall technique?	☐ Complete removal of all carious dentin ☐ Sealing the carious lesion from the oral environment to arrest the disease process ☐ Elimination of all bacteria through disinfection ☐ Strengthening the remaining tooth structure ☐ I am not sure
B3	Which of the following is a PRIMARY indication for the Hall technique? (Select all that apply)	☐ Asymptomatic carious primary molar ☐ Primary molar with pulpal involvement ☐ Conventional restoration not possible due to child behavior ☐ Primary molar with signs of irreversible pulpitis ☐ Non-restorable primary molar requiring extraction
B4	Which of the following are CONTRAINDICATIONS to the Hall technique? (Select all that apply)	☐ History of spontaneous pain ☐ Clinical signs of pulpal pathology (e.g., abscess, sinus tract) ☐ Deep caries approaching the pulp with reversible pulpitis ☐ A primary molar with a well-defined radiolucency ☐ Allergy to stainless steel ☐ None of the above
B5	Do you believe there are any medical contraindications to the Hall technique?	☐ Yes ☐ No ☐ I am not sure
B6	What is the recommended follow-up period for a Hall technique crown?	☐ 3 months ☐ 6 months ☐ 12 months ☐ 24 months ☐ I am not sure

**Table 9:** Section C: Attitudes toward the Hall technique. Instructions: Please indicate your level of agreement with each statement using the following scale: 1 = strongly disagree, 2 = disagree, 3 = neutral, 4 = agree, 5 = strongly agree.

Item	Statement	1	2	3	4	5
C1	I believe the Hall technique is a valid treatment option for managing carious primary molars.	☐	☐	☐	☐	☐
C2	The Hall technique is supported by strong scientific evidence.	☐	☐	☐	☐	☐
C3	The Hall technique is time-efficient compared to conventional restorative procedures.	☐	☐	☐	☐	☐
C4	The Hall technique is cost-effective for patients and healthcare systems.	☐	☐	☐	☐	☐
C5	The Hall technique is a biologically sound approach to caries management.	☐	☐	☐	☐	☐
C6	The Hall technique is best suited for uncooperative pediatric patients.	☐	☐	☐	☐	☐
C7	The Hall technique is acceptable to parents/guardians when properly explained.	☐	☐	☐	☐	☐
C8	I feel confident in my ability to perform the Hall technique.	☐	☐	☐	☐	☐
C9	The Hall technique should be taught as a core skill in pediatric dentistry training programs.	☐	☐	☐	☐	☐
C10	I am interested in receiving additional training on the Hall technique.	☐	☐	☐	☐	☐

**Table 10:** Section D: Clinical practice.

Item	Question	Response options
D1	Are you currently using the Hall technique in your clinical practice?	☐ Yes ☐ No
D2	If YES, approximately what percentage of your carious primary molars do you manage using the Hall technique?	☐ <10% ☐ 10–25% ☐ 26–50% ☐ 51–75% ☐ >75%
D3	If NO, what are the PRIMARY reasons for not using the Hall technique? (Select all that apply)	☐ Lack of training ☐ Concern about sealing over active caries ☐ Concern about parental acceptance ☐ Concern about occlusal issues ☐ Preference for traditional restorative techniques ☐ Lack of access to preformed metal crowns ☐ Other: ________
D4	What are your MAIN concerns regarding the Hall technique? (Select all that apply)	☐ Sealing over active caries ☐ Parental acceptance ☐ Occlusal issues ☐ Long-term success rate ☐ Difficulty in crown selection and fitting ☐ Lack of evidence ☐ Cost of materials ☐ Other: ________
D5	Have you encountered any complications with the Hall technique in your practice? (Select all that apply)	☐ Crown loss ☐ Crown fracture ☐ Caries progression ☐ Pain or discomfort ☐ Occlusal issues ☐ Parental dissatisfaction ☐ No complications experienced ☐ Other: ________
D6	How did you first learn about the Hall technique? (Select all that apply)	☐ Formal postgraduate training ☐ Continuing education courses ☐ Journal articles and scientific literature ☐ Colleagues ☐ Social media ☐ International conferences ☐ Other: ________
D7	How often do you use the Hall technique in your clinical practice?	☐ Daily ☐ Weekly ☐ Monthly ☐ Occasionally ☐ Rarely ☐ Never
D8	Would you be interested in attending a hands-on workshop or continuing education course on the Hall technique?	☐ Yes ☐ No ☐ Possibly

## References

[1] Frencken JE, Peters MC, Manton DJ, Leal SC, Gordan VV, Eden E (2012). Minimal intervention dentistry for managing dental caries - a review: report of a FDI task group. Int Dent J.

[2] Ricketts D, Lamont T, Innes NP, Kidd E, Clarkson JE (2013). Operative caries management in adults and children. Cochrane Database Syst Rev.

[3] Innes NP, Evans DJ, Stirrups DR (2007). The Hall technique; a randomized controlled clinical trial of a novel method of managing carious primary molars in general dental practice: acceptability of the technique and outcomes at 23 months. BMC Oral Health.

[4] Innes NP, Evans DJ, Stirrups DR (2011). Sealing caries in primary molars: randomized control trial, 5-year results. J Dent Res.

[5] Schwendicke F, Stolpe M, Innes N (2016). Conventional treatment, Hall technique or immediate pulpotomy for carious primary molars: a cost-effectiveness analysis. Int Endod J.

[6] Santamaria RM, Innes NP, Machiulskiene V, Evans DJ, Alkilzy M, Splieth CH (2015). Acceptability of different caries management methods for primary molars in a RCT. Int J Paediatr Dent.

[7] Santamaria RM, Innes NP, Machiulskiene V, Evans DJ, Splieth CH (2014). Caries management strategies for primary molars: 1-yr randomized control trial results. J Dent Res.

[8] Roberts A, McKay A, Albadri S (2018). The use of Hall technique preformed metal crowns by specialist paediatric dentists in the UK. Br Dent J.

[9] Alshahrani A, Alamro H, Alanazi F, Alowaidi L, Alhamdan F (2025). Assessment of knowledge, attitude, and teaching of the Hall technique among pediatric dentistry faculty in Riyadh, Saudi Arabia. Dent J (Basel).

[10] Hussein I, Al Halabi M, Kowash M (2020). Use of the Hall technique by specialist paediatric dentists: a global perspective. Br Dent J.

[11] von Elm E, Altman DG, Egger M, Pocock SJ, Gøtzsche PC, Vandenbroucke JP (2008). The Strengthening the Reporting of Observational Studies in Epidemiology (STROBE) statement: guidelines for reporting observational studies. J Clin Epidemiol.

[12] Peduzzi P, Concato J, Kemper E, Holford TR, Feinstein AR (1996). A simulation study of the number of events per variable in logistic regression analysis. J Clin Epidemiol.

[13] Altoukhi DH, El-Housseiny AA (2020). Hall technique for carious primary molars: a review of the literature. Dent J (Basel).

[14] Greenhalgh T, Robert G, Macfarlane F, Bate P, Kyriakidou O (2004). Diffusion of innovations in service organizations: systematic review and recommendations. Milbank Q.

[15] Mirsiaghi F, Leung A, Fine P, Blizard R, Louca C (2018). An investigation of general dental practitioners' understanding and perceptions of minimally invasive dentistry. Br Dent J.

